# Digital Light Processing (DLP) 3D Printing of Atomoxetine Hydrochloride Tablets Using Photoreactive Suspensions

**DOI:** 10.3390/pharmaceutics12090833

**Published:** 2020-08-31

**Authors:** Mirjana Krkobabić, Djordje Medarević, Nikola Pešić, Dragana Vasiljević, Branka Ivković, Svetlana Ibrić

**Affiliations:** 1 Department of Pharmaceutical Technology and Cosmetology, Faculty of Pharmacy, University of Belgrade, Vojvode Stepe 450, 11221 Belgrade, Serbia; mirjana.krkobabic@pharmacy.bg.ac.rs (M.K.); djordje.medarevic@pharmacy.bg.ac.rs (D.M.); nikola.pesic@pharmacy.bg.ac.rs (N.P.); dragana.vasiljevic@pharmacy.bg.ac.rs (D.V.); 2 Department of Pharmaceutical Chemistry, Faculty of Pharmacy, University of Belgrade, Vojvode Stepe 450, 11221 Belgrade, Serbia; branka.ivkovic@pharmacy.bg.ac.rs

**Keywords:** three-dimensional (3D) printing, digital light processing (DLP), photopolymerization, photoreactive suspensions, personalized therapy, sustained release

## Abstract

Three-dimensional (3D) printing technologies are based on successive material printing layer-by-layer and are considered suitable for the production of dosage forms customized for a patient’s needs. In this study, tablets of atomoxetine hydrochloride (ATH) have been successfully fabricated by a digital light processing (DLP) 3D printing technology. Initial materials were photoreactive suspensions, composed of poly(ethylene glycol) diacrylate 700 (PEGDA 700), poly(ethylene glycol) 400 (PEG 400), photoinitiator and suspended ATH. The amount of ATH was varied from 10.00 to 25.00% (*w/w*), and a range of doses from 12.21 to 40.07 mg has been achieved, indicating the possibility of personalized therapy. The rheological characteristics of all photoreactive suspensions were appropriate for the printing process, while the amount of the suspended particles in the photoreactive suspensions had an impact on the 3D printing process, as well as on mechanical and biopharmaceutical characteristics of tablets. Only the formulation with the highest content of ATH had significantly different tensile strength compared to other formulations. All tablets showed sustained drug release during at least the 8h. ATH crystals were observed with polarized light microscopy of photoreactive suspensions and the cross-sections of the tablets, while no interactions between ATH and polymers were detected by FT-IR spectroscopy.

## 1. Introduction

Oral solid dosage forms are manufactured in precisely defined strengths, which can provide therapeutic effects for most of the population. The splitting of a solid dosage form is often necessary for dose adjustment to specific patients, which can result in a variation of drug content and inappropriate therapy. Dosing requirements change in different physiological and metabolic functions, and in comorbidities, often manifested in elders and children [1].

Current pharmaceutical manufacturing is not suitable for personalized medicines, while application of the three-dimensional (3D) printing (an additive manufacturing technology) in pharmacies might enable the production of small customized batches of solid dosage forms for oral administration with different dose and release characteristics [2,3].

All types of 3D printing technologies are based on the successive printing of material in layers, where a desired form predefined by 3D modeling software is obtained at the end of the printing [4]. 3D printing technologies can facilitate the fabrication of different dosage forms containing multiple drugs [5,6,7], dosage forms with immediate drug release [8,9,10], and modified drug release profiles [11,12]. Technologies investigated so far for production of medicines, where different drug releases were achieved, were the most commonly based on material extrusion [13,14], photopolymerization [12,15,16], binder jetting [17] and selective laser sintering (SLS) [18,19]. The main differences between 3D printing processes are the nature of the material used and the characteristics of the obtained products [20].

Photopolymerization processes use liquid photocurable resins, which undergo chemical reactions upon irradiation with light and fabricate solid objects [21]. Two types of additive manufacturing using photopolymers are stereolithography (SLA) and digital light processing (DLP). SLA uses a laser beam to scan the surface of the photopolymer mixture, enabling the fabrication of components with high resolution and good surface quality, while DLP uses a projector to selectively expose and cure an entire cross-sectional slice of the photopolymerizable resin at each given time [22,23,24]. In the consecutive immersion of the platform into the resin tank, the uncured photoreactive mixture crosses over the former cured layer, and in that way, every following layer is polymerized again by light and adheres to the previous layer. The process is repeated until the complete tablets are obtained [25].

Easy availability of DLP printers has made this technology promising, primarily in personalized medicine. It is noteworthy that the DLP technology is capable of fabricating dental models [26], and different implants for drug delivery [27]. The main disadvantage of processes based on photopolymerization is the potential cytotoxicity, caused by the unreacted monomer and photoinitiator residues [28]. Compared to other technologies, in DLP printing high drug loading has not been achieved yet, because it is necessary to provide sufficient content of photopolymer (which automatically reduces the possibility of incorporating an increased amount of other components) to obtain uniform tablets.

The photopolymers used so far in investigations of solid pharmaceuticals forms are poly(ethylene glycol) diacrylate (PEGDA) [12,16] and poly(ethylene glycol) dimethacrylate [12]. PEGDA is a synthetic, hydrophilic, biocompatible, and non-immunogenic material that can polymerize in the presence of photoinitiator and light [29].

Photoreactive solutions were mainly used to obtain solid pharmaceutical forms by 3D printing based on polymerization [11,12,16,30]. In our previous work, photoreactive suspensions with a suspended excipient were used in DLP printing, where the differences in the characteristics of the obtained tablets were noticed, depending on the type of initial photoreactive mixture [16]. However, there were no attempts to incorporate suspended active substances in the photoreactive mixture. Suspensions prepared for photopolymerization based 3D printing must be sufficiently transparent to the light to allow an acceptable cure depth [31]. Matching of the refractive index of medium and suspended phase can enable the preparation of highly loaded suspensions due to the minimization of the Van der Waals attraction forces [32]. It is desirable for photoreactive suspensions to have a viscosity less than 3000 mPa·s for proper flow during the printing, where the viscosity of the initial monomer is important for final viscosity of suspensions [31].

The aim of this study was 3D printing of photoreactive suspensions with different amounts of the solid phase, and characterization of the photoreactive suspensions and obtained tablets. Atomoxetine hydrochloride (ATH) was chosen as an active substance due to the wide range of doses used in therapy (500 micrograms/kg daily–100 mg or 1.4 mg/kg daily), and its administration mostly in the child population, making it an ideal candidate for the development of personalized dosage forms [33]. The effect of the composition of photoreactive suspensions on drug release rates and kinetics, mechanical properties of tablets, and interactions between drug and polymers were also analyzed.

## 2. Materials and Methods

### 2.1. Materials

Different photoreactive suspensions were prepared from poly(ethylene glycol) diacrylate 700 (PEGDA 700, Sigma-Aldrich, Tokyo, Japan), poly(ethylene glycol) 400 (PEG 400, Fagron B.V., Rotterdam, The Netherlands), atomoxetine hydrochloride (ATH, kindly provided by Hemofarm AD, Vrsac, Serbia), and diphenyl (2,4,6-trimethylbenzoyl) phosphine oxide (DPPO, Sigma-Aldrich, Steinheim, Germany). All other chemicals and reagents used in the study were of analytical grade.

### 2.2. Methods

#### 2.2.1. Preparation of Photoreactive Suspensions

ATH was used as an active substance whose content varied from 10.00% to 25.00% (*w/w*), while PEGDA was used as a photopolymer and DPPO as a photoinitiator. In photoreactive suspensions, PEG 400 was used as an excipient to overcome very slow and incomplete drug release from tablets fabricated by technologies based on photopolymerization. The ratio of PEGDA and PEG 400 was constant in all formulations (3:1) (Table 1).

PEG 400 was chosen due to previous experience, where it was used in the production of uniform tablets, and PEGDA/PEG 400 ratio 3:1 was used for the production of the tablets with appropriate tensile strength and dissolution rate [16]. It was prepared 50 g of each photoreactive suspension by mixing on the magnetic stirrer for 12 h. During the preparation and the printing process, all formulations were protected from the light.

#### 2.2.2. Determination of Drug Content in Photopolymer Suspensions

The drug content in photopolymer suspensions of each formulation was determined in triplicate. Photopolymer suspensions (quantity equivalent to 10 mg of active substance) were placed in a volumetric flask with distilled water (100 mL) and shaken on the ultrasound bath for 15 min. The samples then were filtered through 0.45 µm filters (Millipore, Bedford, MA, USA), and drug loading was determined by UV/VIS spectrophotometry (Evolution 300, Thermo Fisher Scientific, Waltham, MA, USA) at 270 nm.

#### 2.2.3. Determination of Drug Content in Tablets

Three separate tablets were crushed per batch and 10 mg equivalents taken from the one tablet each, dissolved in 100 mL of distilled water and shaken inside the ultrasound bath for 15 min. The samples then underwent the same procedure as described for samples prepared from photopolymer suspensions (Section 2.2.2). The results were expressed as an average ATH ± standard deviation (S.D.) [34].

#### 2.2.4. Refractive Index of the Suspension Measurements

The refractive index *n* of all photoreactive suspensions was determined using a refractometer (ABBE bench refractometer, 2WAJ, OPTIKA microscopes, Ponteranica, Italy). The refractometer was set up to measure the refractive index in the wavelength corresponding to the D-line of sodium (589.3 nm). An average of three measurements was taken for all photoreactive suspensions.

#### 2.2.5. Rheological Measurements

The rheological measurements of the suspensions were carried out on a Rheolab MC 120 (Paar Physica, Stuttgart, Germany) rheometer coupled with the rotating cylinder measuring device Z3 DIN (diameter 25 mm) at 20 ± 0.1 °C. The controlled shear rate (CSR) procedure was applied to the flow curve construction, by increasing the shear rate from 0 to 200 1/s. The rheological characterization of the photoreactive suspensions was performed to relate the properties of the photopolymer mixtures via the 3D printing process and the characteristics of the obtained tablets. The measurements were performed in triplicate for each sample.

#### 2.2.6. 3D Printing of ATH Tablets

**Figure 1:**
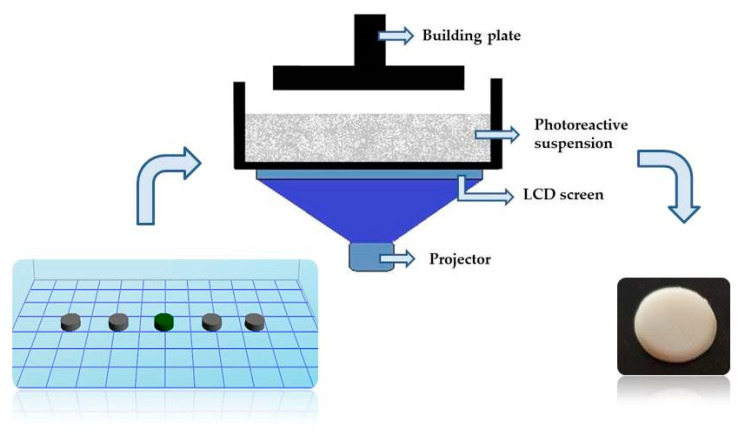
Illustration of the 3D DLP printing.

The cylindrical shape of the tablets was designed by Autodesk Fusion 360 software (Autodesk Inc., San Rafael, CA, USA) and exported as a stereolithography file (.stl) into Creation Workshop X 1.2.1 software. The DLP printer Duplicator 7 (Wanhao, Zhejiang, China) (Figure 1) was used for the fabrication of tablets for all formulations. The dimensions of tablets were 8 mm in diameter and 2 mm in height.

For formulations A1–A4, it was examined which printing parameters allow the adhesion of the tablets to the building plate and do not lead to over curing of the photoreactive suspension. Previous studies [15,35] have shown that it is necessary to adjust the printing parameters for each formulation separately, because there are no predefined optimal conditions as is the case with commercially available photoreactive mixtures used in other industries. Photopolymerization was conducted for layers thickness of 0.1 mm, to prevent problems with light penetration which can occur due to the interaction of light with suspended particles. All tablets were fabricated including 5 bottom layers, with different bottom layer exposure for different formulations, depending on the proportion of ATH in photoreactive suspensions. For tablets containing 10.00% and 15.00% of ATH, the bottom layer exposure was set at 30 s, while for tablets containing 20.00% and 25.00% of ATH was set at 70 s. Higher bottom layer exposure was required for tablets with a higher proportion of active substance to adhere to the building plate. The exposure time of the remaining layers was 15 s per layer for each formulation, and the total number of layers, following the specified dimensions, was 20. The printing time depended on the total exposure time and lasted from 10 min 30 s to 13 min 15 s for 5 tablets.

#### 2.2.7. Determination of Mass, Dimension and Tensile Strength of Tablets

Mass was determined on 20 tablets, while dimensions (digital caliper, Vogel Germany GmbH & Co. KG, Kevelaer, Germany) were measured on 10 tablets for each formulation. Hardness was estimated on 10 tablets per formulation using a hardness tester (TBH 125D, Erweka, Langen, Germany), and tensile strength was calculated according to the following equation [36]:

σ_x_ = 2F/πDt (1)

In the previous equation, σ_x_ is the tensile strength, F is the tablet breaking force, D is the diameter, and t is the thickness of tablets.

Statistical analysis was performed using PASW Statistics 18.0 (SPSS Inc., Chicago, IL, USA), and a comparison of all raw data involved a normality and homogeneity test. A parametric one—way ANOVA was used when homogeneity and normality were found, with significance levels *p* < 0.05. Post hoc Tukey’s test was applied due to the same number of data in every group to determine between which formulations there was significant difference.

#### 2.2.8. Polarized Light Microscopy

The Olympus BX51-P polarizing microscope (Olympus, Tokyo, Japan) with cellSens Entry software was used for visual analysis of ATH powder, photoreactive suspensions and prepared tablets. The particle size of the pure ATH and of the particles in photoreactive suspensions was estimated using the ImageJ software (National Institutes of Health, Bethesda, MD, USA). The photoreactive suspensions were diluted with n-hexane (1:1) to make the ATH particles more visible. Measurements of length were made per one hundred randomly selected particles in diluted photoreactive suspensions, as well as for ATH powder, and the results were shown as mean (±S.D.), minimum, and maximum length.

Polarized light microscopy was used for observation of a cross-section of tablets to compare the internal structure of the tablets with the photoreactive suspensions from which they were obtained and also for the detection of the crystalline components.

#### 2.2.9. Fourier Transform Infrared Spectroscopy (FT-IR)

Nicolet iS10 (Thermo Scientific, Waltham, MA, USA) FT-IR spectrometer, equipped with a single reflection ATR system (Smart iTR, Thermo Scientific, Waltham, MA, USA) with diamond plate and ZnSe lens, was used to record FT-IR spectra of raw materials and crushed tablets. The wavenumbers were from 650 to 4000 cm^−1^, with a resolution 4, while the spectra were collected as an average of 16 scans. The analysis was performed to identify potential interactions between the components in the tablets.

#### 2.2.10. In Vitro Drug Release Testing

Initial experiments performed on 3D printed ATH tablets in our laboratory showed that the choice of apparatus (paddle apparatus or flow-through cell) and medium (distilled water or 0.1 M HCl) had no significant effect on the dissolution rate of ATH from tablets. ATH dissolution rate from 3D printed tablets was tested using USP IV (Flow-through cell, CE7 smart, Sotax, Aesch, Switzerland) apparatus. Three tablets of each formulation were tested in 250 mL of distilled water at 37 ± 0.5 °C, with a flow rate of 8 mL per minute, during 8 h. The samples (4 mL) were withdrawn at 15, 30, 45, 60, 120, 180, 240, 300, 360, 420, and 480 min time intervals and immediately replaced with a fresh medium, while samples were filtered through 0.45 µm membrane filters (Millipore, Bedford, MA, USA). The amount of dissolved ATH was determined by UV/VIS spectrophotometry at 270 nm (Evolution 300, Thermo Fisher Scientific, Waltham, MA, USA).

#### 2.2.11. Kinetic Modeling of Drug Release

The mechanism of ATH release from the tablets was analyzed by fitting the data of an eight-hour in vitro dissolution test of each formulation into zero-order, first-order, Higuchi, and Korsmeyer-Peppas models [37,38]. Fitting the data into these mathematical models was performed by using the DDSolver software (Microsoft Excel add-in program) developed by Zhang et al. [38]. Mathematical models applied to the obtained data are shown in Table 2.

## 3. Results and Discussion

### 3.1. Drug Content in Photopolymer Suspensions and Tablets

The drug content in the different photoreactive suspensions indicates that suspensions were homogenous. The content of ATH in 3D printing tablets and the photoreactive suspensions were similar; only formulation A1, with the lowest content of ATH, had the complete recovery in photoreactive suspension, but all photoreactive suspensions had ATH content 95.58–100.29%, which can be considered as acceptable; the lower content in tablets compared to the content in photoreactive suspensions may be due to the entrapped small fraction of the active substance in the tablet matrix, which is difficult to extract during the sample preparation. It has been previously shown that decrease of drug content in tablets compared to initial mixture could be due to retention of an active substance in the drug-polymer matrix [11]. In all tablets, the content of ATH was 95.35–96.46% of the theoretical content, which indicates that homogeneity in tablets was achieved, and there was no unaccepted degradation of the active ingredient during photopolymerization in the 3D printing process (Table 3).

### 3.2. Refractive Index of The Suspensions Measurements

Measured values of the refractive index are shown in Table 4. All formulations with the ATH had a higher refractive index compared to the placebo formulation (PEGDA + PEG 400 + photoinitiator).

The changed refractive index of photoreactive suspensions compared to the placebo formulation had the potential impact on the printing process, and tablets characteristics. The refractive index of ATH is 1.552 [39], indicating a large difference between the solid phase and placebo refractive indices, which could lead to inaccuracies in printing and differences in dimensions [40], and consequently on the mass of the tablets.

### 3.3. Rheological Characterization of Photoreactive Suspensions

**Figure 2:**
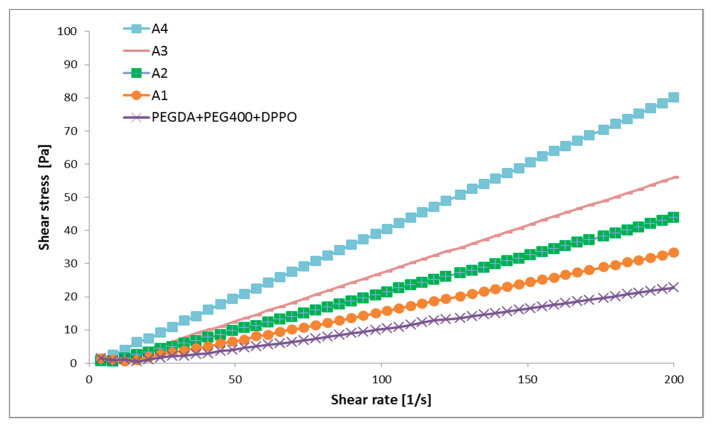
Flow curves of the placebo and the ATH suspensions.

**Figure 3:**
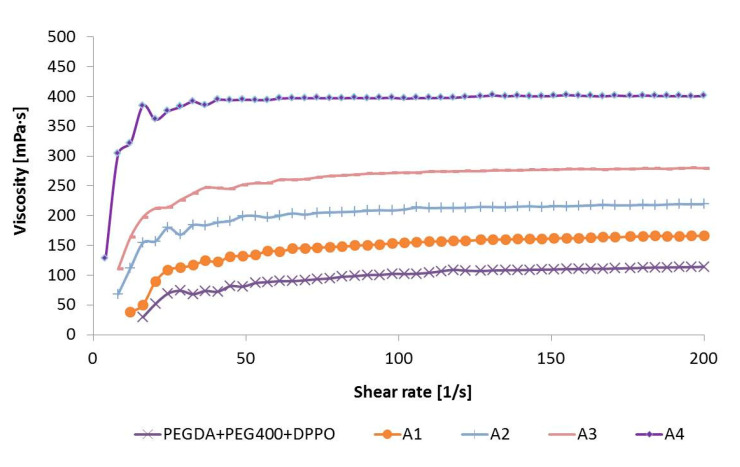
Viscosity curves of the placebo and ATH suspensions.

Flow and viscosity curves of investigated samples are given in Figure 2 and Figure 3, respectively. Placebo and four photoreactive suspensions with ATH exhibited dilatant flow behavior at lower shear rates (below approximately 30 1/s), followed by Newtonian flow at higher shear rates. In dilatant (i.e., shear thickening) flow behavior, the viscosity increases with the increasing shear rate, while a Newtonian fluid’s viscosity remains constant i.e., viscosity is independent of shear rate [41]. The type of flow was the same for placebo and formulations with ATH, so it was most affected by polymers, in the first place by PEGDA.

It is known that the viscosity of suspensions depends on a number of factors, including the volume fraction of the solid phase, the type of suspended particles, and their shape, size and size distribution [42]. In this case, the viscosity of the suspensions increased significantly with the increase in the proportion of ATH. The suspension with the highest proportion of ATH (25.00%) had the highest value of viscosity (approximately 400 mPa·s, at 30–200 1/s), while placebo formulation the lowest viscosity values (approximately 100 mPa·s, at 30–200 1/s) (Figure 3).

All photoreactive suspensions had a viscosity notably bellow 3000 mPa·s (Figure 3), which was important for successful printing [31]. The viscosity of the suspensions affected the printing time, mass, dimensions and tensile strength of the tablets. The high viscosity could hinder precise layer thickness and increase curing time [43].

### 3.4. Polarized Light Microscopy of the ATH Powder and Photoreactive Suspensions

**Figure 4:**
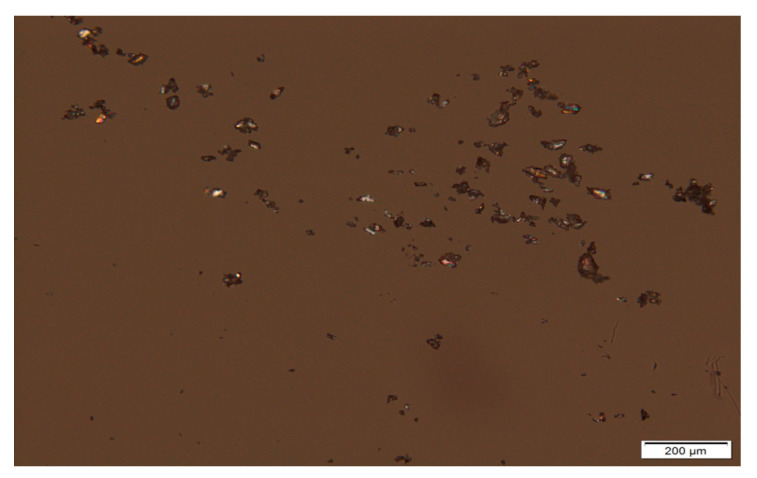
Micrograph of ATH powder sample at 10× magnification.

Particles of pure ATH, mean diameter, the minimal and maximal size of particles, were analyzed on magnification 10× (Figure 4). It is important to emphasize that manually selecting only 100 particles gives a low-quality estimate of the real particle size distribution, but microscopic analysis provides insight into the shape of the particles. The mean particle size was 26.97 ± 12.13 μm, while the minimal and maximal size of particles in the sample was 8.70 and 76.67 μm, respectively, which indicates that there was a wide particle size distribution in the initial powder, and the same could be expected for prepared suspensions.

**Figure 5:**
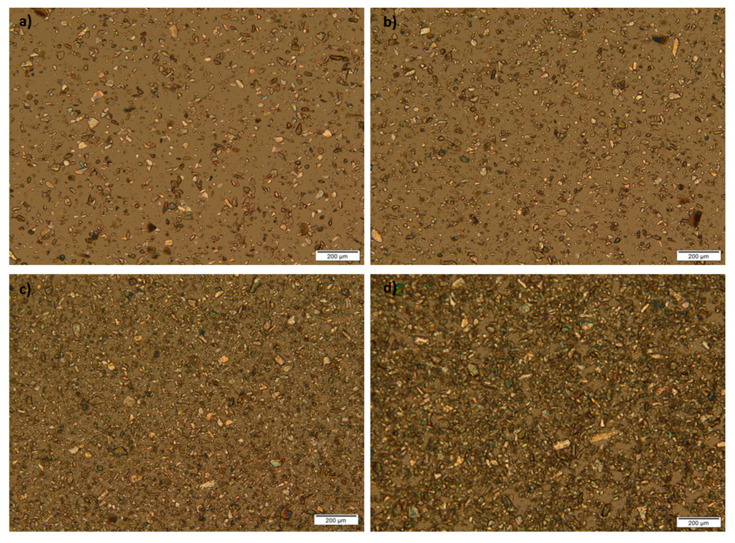
Micrographs of photoreactive suspensions at 10x magnification: (**a**) A1; (**b**) A2; (**c**) A3; (**d**) A4 formulation.

Photoreactive suspensions of ATH were diluted with n-hexane (1:1), to allow better observation of particles that were not dissolved in the polymer mixture. A 10× magnification was used for the observation of the shape and size of particles in photoreactive suspensions. From these micrographs, it can be seen that birefringence occurred; crystals were presented in the samples, and their shapes were irregular (Figure 5). Mean diameter, minimal and maximal size of particles are shown in Table 5.

The particle size distribution was wide, but regardless of that, the production of tablets was possible. It would be necessary to uniform the particle size distribution of the solid phase before the preparation of photoreactive suspensions to obtain results with less variation in mass and dose.

The solubility of ATH over a biologically relevant pH range is all well above the maximum dose strength (100 mg)/250 mL, demonstrating the high solubility of ATH. Its solubility is 27.8 mg/mL in water [44], but ATH has a very bitter taste [45], which can be overcome by preparing the suspension of ATH in PEGDA photopolymer mixture, while the instability of the suspensions can be overcome by using them for tablet preparation, as the final pharmaceutical dosage form [46].

Ideal suspension formulations should contain insoluble particles uniformly suspended in the continuous phase, but during the standing, the solid particles in suspensions get separated from the liquid as sediments. Despite the amount of sedimentation, suspensions should be redispersed uniformly after careful shaking. It is known that the disadvantage of suspension dosage forms includes the possibility of dose variation. The most important parameter connected to the sedimentation is the particle size, where smaller particles yield a low rate of sedimentation. A narrow particle size distribution is desirable for uniform sedimentation rate and then is possible to provide better predictability of suspension properties. Increasing viscosity of the medium can decrease the rate of sedimentation, while the redispersion can be controlled by using flocculating agents. All the mentioned factors affect the stability of suspensions [46]. Photoreactive suspensions differ from suspensions intended for direct application; they represent an initial mixture for 3D printing, however, the same factors should be considered for them. The photoreactive suspensions of ATH were stable enough for the printing process because they were immediately placed in the printer after mixing, where printing took a short time, which is one of the benefits of this type of 3D printing. However, further testing would be needed for a potential production in pharmacies, as the wide particle size distribution and irregular particle shape shown in the micrographs could cause suspension instability and further problems in the printing process.

### 3.5. 3D Printing Process

So far, PEGDA 575, without the addition of any excipients or with addition of PEG 300, was used for printing polypills containing: paracetamol, naproxen, caffeine, aspirin, prednisolone, and chloramphenicol [15], while PEGDA 400 was used for printing tablets containing theophylline as active substance. Paracetamol [11,16], 4-aminosalicylic acid [11] and ibuprofen [35,47], were used as active substances in photopolymerization based printing processes, combined with a mixture of PEGDA and PEGs of different molecular weights. In our study, tablets containing four different amounts of ATH suspended in PEGDA/PEG 400 mixture were successfully produced by the DLP printer. In the work by Xu et al. (2020), PEGDA 575 and PEG 300 were used for production of the antihypertensive polyprintlet (irbesartan, atenolol, hydrochlorothiazide and amlodipine), where chemical reaction between a photopolymer and amlodipine reported [48]. Accordingly, different active substances could be dissolved or suspended in the PEGDA/PEGs mixtures, but it is important to investigate potential interactions between drugs and polymers.

To provide quality and safety of obtained tablets, 3D printing processes for pharmaceutical use need to be complementary with Good Manufacturing Practice (GMP) pharmaceutical standards. In accordance with the described procedure of preparation of the photoreactive suspensions and printing of tablets with DLP 3D technology, this process, as well as other 3D printing technologies, could take a role in preparations of personalized dosage forms in community and hospital pharmacies [49]. The benefits of using the DLP printer here demonstrated are the possibility of customization of the printing parameters depending on the formulation factors, fast printing of pharmaceutical dosage forms, as well as printing at room temperature. Previous studies based on DLP 3D printing have also shown these possibilities as potential advantages of this technology [12,16,35]. In comparison with other 3D printing technologies investigated for tablet production, DLP has a limited number of photopolymers suitable for pharmaceutical use.

### 3.6. Appearance, Mass, Doses, and Dimension of the Tablets

Tablets of all formulations were white and showed the same color as the initial photoreactive suspensions. Mass, dose, diameter, and thickness of the tablets are shown in Table 6.

The increase in the ATH content led to the fabrication of tablets with a higher mass and dimensions, due to the higher viscosity, and the higher proportion of ATH particles suspended and their scattering phenomena in the light beam. With increasing content of particles in the suspensions, the intensity of light scattering increases and affects the dimensions and consequently the mass of the tablets; when the particle size is not constant (as in the case of A1–A4 formulations), it is not possible to establish a linear relationship between particle concentration and light scattering [50]. It has been shown by Mitteramskogler and Gerald (2014) that the light scattering in ceramic suspensions caused a certain amount of widening of the dimensions in the final geometry, where exposure time and exposure area also had influence [51]. Printing from photoactive solutions did not cause problems with the dimensions and the shape of the tablets, as shown in the works of Kadry et al. (2019) and Martinez et al. (2018) [12,30], while photoreactive suspensions can cause over curing during the printing tablets of different shapes and sizes [16], which was avoided in this research by production tablets of smaller dimensions (8 mm in diameter). According to “Guideline on pharmaceutical development of medicines for pediatric use”, tablets smaller than 10 mm are suitable for use in children aged above 6 years [52], which indicates that ATH 3D printed tablets could be used in the target population because ATH is used in the treatment of attention deficit hyperactivity disorder in adults and children aged 6 years and over [33].

Formulation A3 had the smallest variation from the previously set diameter, while formulation A2 varied the least from the thickness specified in the Creation Workshop software. Formulation A1 had a diameter and a thickness less than the specified, while formulation A2 had only a diameter lower than the specified 8 mm. Formulations A3 and A4 had dimensions larger than previously set. ATH tablets had smaller deviations from the given dimensions compared to the tablets containing suspended mannitol in the previous study. However, tablets containing mannitol had longer exposure time conditions and over curing was a possibility that could occur, which was not the case with ATH tablets [16].

The achieved dose was from 12.21 to 40.07 mg, which can allow personalization of the therapy. The standard deviation that occurred in the variation of the tablet’s mass and thus the achieved dose could be reduced by using a higher resolution 3D printer and a uniform particle size in the suspension.

### 3.7. Tensile Strength of the Tablets

The ratio of PEGDA and PEG 400 was the same in all formulations, so the content of ATH and amount of photoinitiator relative to PEGDA: PEG 400 influenced the tensile strength of the tablets. Increasing content of active ingredient led to the higher tensile strength of tablets, and all formulations had tensile strength around 1 MPa. Although 1 MPa is appropriate for the production of small batches [53], due to the operating with the photoreactive suspensions and the printer, the achieved tensile strengths may be sufficient for use in individual patients in pharmacies.

**Figure 6:**
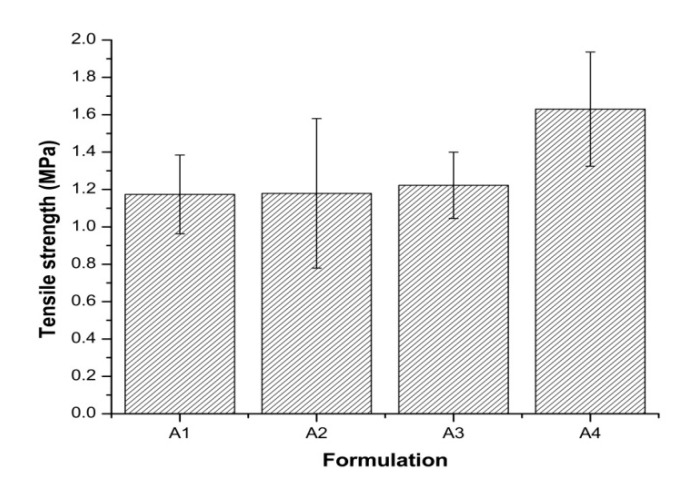
Tensile strength of all formulations.

Only formulation A4 shows a statistically significant difference compared to other formulations. It was possible to print tablets with 10.00%, 15.00%, and 20.00% of ATH without significantly affecting the mechanical properties of the tablets (Figure 6). Increasing tensile strength of tablets with increasing the proportion of ATH can also be the result of different bottom layer exposures, where longer bottom layer exposure can lead to better adhesion of the layers.

High variations of tablet tensile strength were observed in all formulations which may be due non-uniform photopolymerization in all directions of the tablet layers, which can be caused by the wide range of particle size distribution.

### 3.8. Polarized Light Microscopy of the Tablets Cross-Section

**Figure 7:**
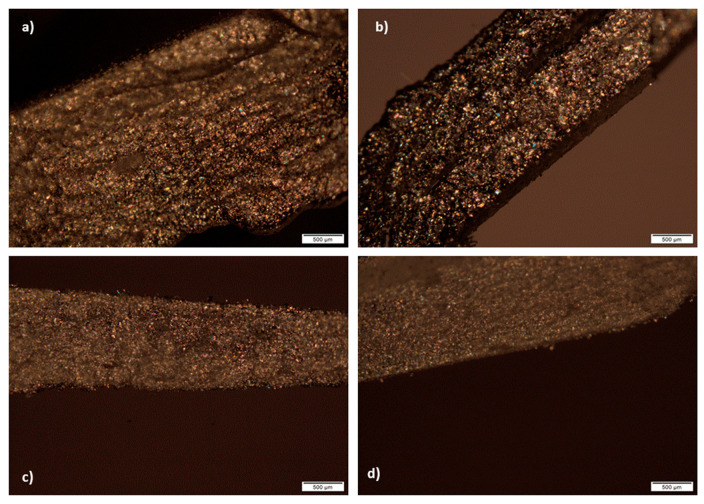
Cross-sectional views of tablets: (**a**) A1; (**b**) A2; (**c**) A3; (**d**) A4 formulations.

In all cross-sectional views of 3D printed tablets containing 10.00–25.00% of suspended ATH, visible parallel layers cannot be observed. It can be seen on micrographs that birefringence occurs and that in tablets, as well as in photoreactive suspensions, crystals were presented (Figure 7). In this way, it has been shown that ATH remained in crystalline state in tablets. It is important to emphasize that homogenous distribution of ATH in the tablets was observed, without agglomerates. Nonhomogeneous distribution of drugs, with agglomeration of drug particles can cause unacceptable variations in drug release within the same batch of extended release matrix tablets. Darker structures on the micrographs most likely appeared due to thicker parts of the tablets cross-section, which disabled light penetration and could be seen during the analysis.

As shown in a previous study, it was possible to see the layers on the cross-section of the tablets obtained from the photoreactive solutions in DLP printing using polarized light microscopy, while the presence of suspended ingredients prevents observation of parallel layers [16]. This indicates that in the case of ATH tablets, the layers were not visible due to the ATH crystals that covered them.

### 3.9. Fourier Transform Infrared Spectroscopy (FT-IR)

FT-IR spectroscopy was used for the detection of interactions between ATH and polymers used for the preparation of tablets.

**Figure 8:**
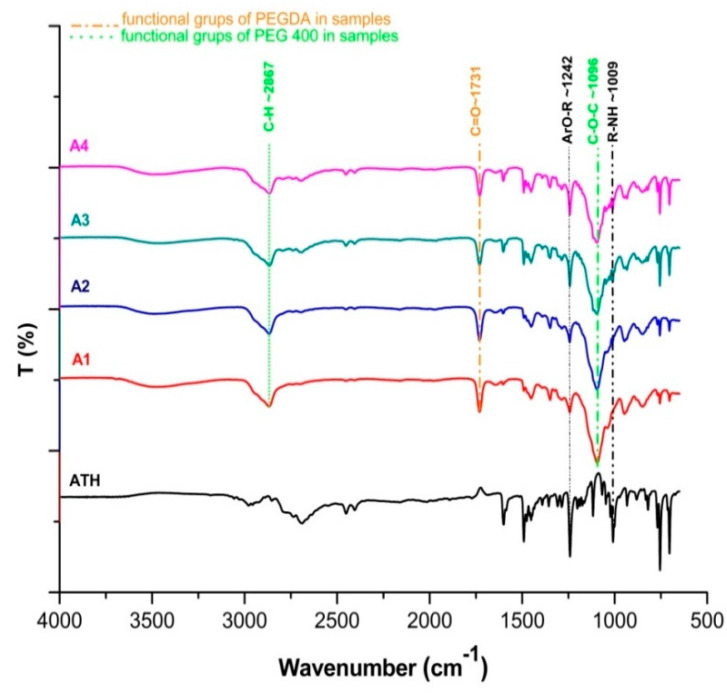
FT-IR spectra of pure ATH and A1-A4 formulations.

Liquid uncured PEGDA contained characteristic acrylate peaks at ∼1636 cm^−1^ and ∼1618 cm^−1^ (C=C stretching) [54], which was difficult to identify in A1-A4 formulations after photopolymerization, due to conversion of C=C to C–C bonds [16,55]. The PEGDA showed characteristic C=O symmetric stretching absorption band at 1731 cm^−1^ in all ATH formulations [56], while peaks characteristic for the PEG 400 were at ~1096 cm^−1^ (C–O–C ether stretching) and at ~2867 cm^−1^ corresponding to –C–H symmetric and asymmetric stretching vibrations [57]. Prominent peaks from ATH observed in tablets were at ~1242 and ~1009 cm^−1^ indicating ArO-R stretching and R-NH stretching [58]. It has been shown that there was a negligible difference in the position of the peaks and there were no drug-polymer interactions (Figure 8).

### 3.10. In Vitro ATH Release

**Figure 9:**
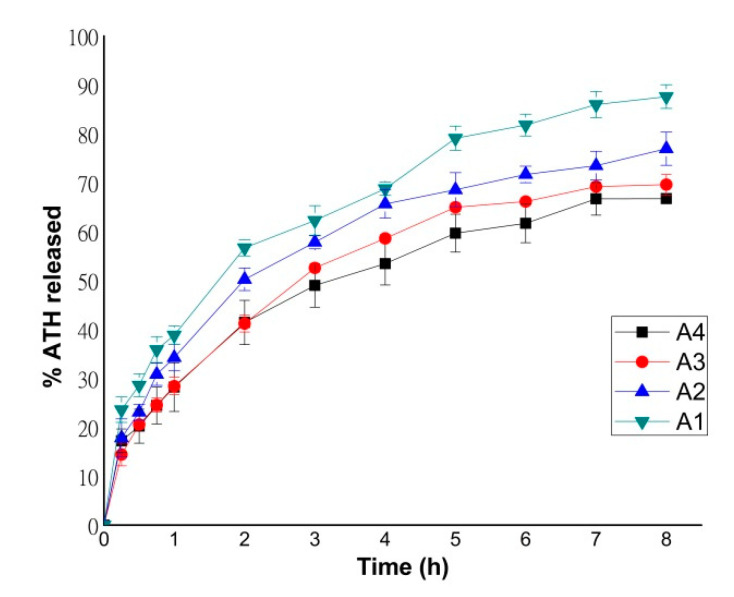
Dissolution profiles of formulations A1–A4.

Dissolution profiles of formulations containing different amounts of ATH are shown in Figure 9. After 8 h, 66.75% and 69.62% of ATH was released from formulations A1 and A2, while 76.96% and 87.60% of ATH was released from formulations A3 and A4, respectively. As previously shown, PEGDA polymer has been confirmed to be suitable for the production of sustained release tablets [11,16,30].

The content of ATH in formulations influenced the dissolution rate; with the increasing of the ATH content, the percentage of the released substance also increased during 8 h. Formulation A4 had the lowest content of PEGDA, and this is a potential reason why this formulation had the fastest drug release. As shown in previous studies, a lower amount of PEGDA probably increased the drug release rate due to a lower degree of cross-linking in the tablet matrix [11,16].

During dissolution, the ATH tablets did not suffer erosion from the surface or disintegration into smaller fractions, but swelling occurs and the tablets remained intact at the bottom of the cell, which was demonstrated in previous studies where PEGDA was used as a photopolymer [12,15,54]. Tablets printed from higher viscosity suspensions showed a higher dissolution rate after 8 h.

### 3.11. ATH Release Kinetics

Model dependent analysis of the obtained ATH release profiles was used to elucidate the impact of increasing content of the suspended active substance on the drug release. It has been previously shown that the mechanism of drug release from formulations with PEGDA and PEG 400 is most consistent with the Korsmeyer-Peppas model, as well as for formulations where mannitol and sodium-chloride solution were used as excipients [16]. In the previous study [35] where, in addition to PEGDA and PEG 400, water was used as an additional excipient, most formulations showed Higuchi kinetics, while some formulations also showed zero-order kinetics. In the study by Cerda et al. (2020), it was shown that in FDM technology, in which PVA, PLA, and PVA/polyethylene glycol were used as polymers, obtained tablets also showed Korsmeyer Peppas kinetics [59]. Mechanism of ATH release from the 3D DLP obtained tablets was best fitted with the Korsmeyer-Peppas model, and increasing of ATH content did not influence drug release kinetics (Table 7).

All formulations had release exponent (*n*) below 0.45, which indicates that ATH was released from tablets dominantly by following the Fickian diffusion mechanism [60]. It was expected that the change in the content of active substance did not affect the release kinetics, only the dissolution rate.

## 4. Conclusions

The possibility of successful 3D DLP printing, using the active substance suspended in the photopolymer mixture, for printing tablets in therapeutic doses for personalized therapy has been demonstrated. The highest content of the active substance (up to 25%) using DLP technology has been incorporated in tablets so far, with flow properties and viscosity values of photoreactive suspensions suitable for 3D printing. Tablets with a higher amount of suspended ATH had shown an increased drug release rate after 8 h, with increasing tensile strength, mass, and dimensions. All formulations showed Korsmeyer-Peppas kinetics after data fitting. Polarized light microscopy confirmed the presence of ATH crystals both in photoreactive suspensions and in the cross-sections of the tablets, while FT-IR analysis showed that there were no interactions between the active substance and other matrix components. All the obtained results indicated the expansion of the possibility of applying 3D printing techniques based on photopolymerization for pharmaceutical purposes. The application of this type of additive manufacturing provides an opportunity for quick and easy personalization of therapy, and all research with the presented advantages and disadvantages of DLP printing can indicate the direction of further development for potential application in pharmacy.

## Figures and Tables

**Table 1:** Different photoreactive suspensions for 3D printing.

	Substance	ATH (% *w/w*)	PEGDA (% *w/w*)	PEG 400 (% *w/w*)	DPPO (% *w/w*)
Formulation					
**A1**		10.00	66.75	22.25	1.00
**A2**		15.00	63.00	21.00	1.00
**A3**		20.00	59.25	19.75	1.00
**A4**		25.00	55.50	18.50	1.00

**Table 2:** Equations of mathematical models commonly used to describe mechanisms of drug release, where Mt/M∞ is the fraction (%) of drug released after time t, K_0_, K_1_, K_H_, K_P_ are drug release rate constants for the corresponding models and n is release exponent, indicating the mechanism of transport of drug.

Mathematical Models	Equations
Zero-order	M_t_/M_∞_ = K_0_∙t
First-order	M_t_/M_∞_ = 100 [1 − Exp(−K_1_∙t)]
Higuchi model	M_t_/M_∞_ = K_H_∙t^1/2^
Korsmeyer-Peppas model	Mt/M_∞_ = K_p_∙t^n^

**Table 3:** Drug content in photopolymer suspensions and tablets (% ± S.D.).

Formulation	Drug Content in Photoreactive Suspensions	Drug Content in Tablets
**A1**	100.29 ± 0.87	96.46 ± 3.88
**A2**	95.66 ± 1.88	95.62 ± 2.93
**A3**	96.78 ± 0.90	96.12 ± 1.80
**A4**	95.58 ± 1.07	95.35 ± 0.70

**Table 4:** Refractive index of the placebo and the ATH formulations (mean value ± S.D.).

Formulation	Refractive Index
**PEGDA+PEG 400 + photoinitiator**	1.4572 ± 0.0000
**A1**	1.4661 ± 0.0001
**A2**	1.4675 ± 0.0004
**A3**	1.4685 ± 0.0004
**A4**	1.4690 ± 0.0001

**Table 5:** Mean diameter ± S.D., minimal and maximal size of ATH particles in photoreactive suspensions.

Formulation	Mean ± S.D. (μm)	Min (μm)	Max (μm)
**A1**	22.60 ± 12.57	4.36	63.65
**A2**	21.53 ± 10.14	5.29	58.50
**A3**	29.61 ± 12.51	8.61	71.84
**A4**	34.68 ± 13.27	13.61	72.93

**Table 6:** Mass, dose and dimensions of the tablets (mean ± S.D.).

	Parameter	Mass ± S.D. (mg)	Dose ± S.D. (mg)	Diameter ± S.D. (mm)	Thickness ± S.D. (mm)
Formulation					
**A1**		122.08 ± 11.69	12.21 ± 1.70	7.70 ± 0.17	1.94 ± 0.17
**A2**		130.18 ± 10.00	19.53 ± 1.50	7.84 ± 0.18	2.03 ± 0.13
**A3**		159.81 ± 11.35	31.96 ± 2.27	8.06 ± 0.30	2.07 ± 0.23
**A4**		160.29 ± 4.71	40.07 ± 1.18	8.35 ± 0.13	2.16 ± 0.08

**Table 7:** ATH drug release kinetics.

Formulation	Model	R^2^ Adjusted	*n* Value
**A1**	Korsmeyer-Peppas	0.9893	0.413
**A2**	Korsmeyer-Peppas	0.9854	0.437
**A3**	Korsmeyer-Peppas	0.9787	0.395
**A4**	Korsmeyer-Peppas	0.9944	0.388
